# Early Postpartum Change in Lactoferrin in Bovine Colostrum During the First 12 h Postpartum and Its Relationship with On-Farm Quality Indicators

**DOI:** 10.3390/vetsci13030293

**Published:** 2026-03-20

**Authors:** Elena Stancheva, Aneliya Milanova, Toncho Penev, Gergana Bachevska, Dimo Dimov

**Affiliations:** 1 Department of Ecology and Animal Hygiene, Faculty of Agriculture, Trakia University, 6000 Stara Zagora, Bulgaria; tonchopenev@abv.bg (T.P.); gergana.iotova@trakia-uni.bg (G.B.); dimo.p.dimov@trakia-uni.bg (D.D.); 2 Department of Pharmacology, Animal Physiology, Biochemistry and Chemistry, Faculty of Veterinary Medicine, Trakia University, 6000 Stara Zagora, Bulgaria; aneliya.milanova@trakia-uni.bg

**Keywords:** bovine colostrum, lactoferrin, Brix refractometer, colostrum specific gravity, LC–MS/MS, passive immunity, neonatal calf

## Abstract

This pilot study aimed to evaluate the early postpartum dynamics of lactoferrin concentration in bovine colostrum and to investigate its relationship with rapid on-farm quality indicators during the first 12 h after calving. Colostrum samples were collected from six multiparous cows immediately after calving (0 h) and again 12 h later. Colostrum specific gravity and % Brix values were measured on-farm, and lactoferrin concentration was determined using LC–MS/MS analysis. Temporal changes were assessed using the Wilcoxon signed-rank test, and relationships between variables were evaluated using linear regression and Spearman correlation analysis. Lactoferrin concentration decreased significantly between 0 and 12 h after calving (median: 3.350 vs. 2.175 mg/mL; *p* = 0.031). In parallel, statistically significant decreases were observed in both colostrum specific gravity and % Brix values over the same period (*p* = 0.031 for both indicators). Linear regression analyses showed positive slopes between lactoferrin concentration and specific gravity and between lactoferrin concentration and % Brix at both 0 and 12 h after calving; however, these relationships did not reach statistical significance (*p* > 0.05 for all models), with explained variance ranging from R^2^ = 0.156 to 0.409. Spearman correlation analysis also revealed moderate positive correlation coefficients (*p* > 0.05), although none of the correlations reached statistical significance. These results indicate a rapid decline in lactoferrin concentration during the first 12 h after calving, occurring in parallel with significant decreases in widely used on-farm colostrum quality indicators. Despite the lack of statistically significant associations, the observed positive relationships indicate that lactoferrin may represent an additional component of colostrum composition that is not directly reflected by refractometric and density-based indicators.

## Simple Summary

Colostrum is essential for immune protection in newborn calves. Farm-based tests are widely used to assess colostrum quality, but they mainly reflect antibody levels and may not capture other important immune proteins. This study examined how lactoferrin concentration in bovine colostrum changes during the first 12 h after calving and how it relates to common farm quality indicators. Lactoferrin levels decreased rapidly over this period and showed positive but non-significant relationships with standard colostrum quality measures. These results suggest that lactoferrin reflects additional aspects of colostrum immune quality not fully captured by routine farm tests.

## 1. Introduction

Colostrum is the first and most important source of passive immunity and bioactive components for newborn calves and plays a major role in survival, health, and long-term productivity. Good management of the colostral period is essential for effective transfer of passive immunity, and colostrum quality largely determines the concentrations of immunoglobulins and other functional proteins [1,2,3]. Because colostrum composition varies greatly between cows and across lactations, routine evaluation of colostrum quality is widely recommended in veterinary practice [3,4]. On farms, rapid methods such as Brix refractometry (% Brix) and measurement of specific gravity using a colostrometer are commonly used as indirect indicators of immunoglobulin G (IgG) concentration [5,6]. Survey studies conducted in different dairy production systems in North America and Europe have documented the use of these tools by farmers as part of routine colostrum management and quality assessment practices [7,8,9]. These methods are well validated and widely used as practical and cost-effective tools for quick assessment of colostrum quality [3,4,10,11]. In addition to immunoglobulins, colostrum contains many bioactive proteins and peptides that support innate immunity, regulate inflammation, and contribute to the development of the gastrointestinal and immune systems in the newborn calf. Lactoferrin is one of the best-studied functional proteins in colostrum and has well-known antimicrobial, immunomodulatory, and anti-inflammatory effects [12,13]. Recent studies have quantified lactoferrin concentrations in bovine colostrum and examined their relationships with other milk components, highlighting the complexity of bioactive protein composition during the transition from colostrum to mature milk [14]. It is involved in iron metabolism, limits the growth of pathogenic microorganisms, and influences cellular immune responses, highlighting its role in innate immune defense. Recent studies show that colostrum composition, including bioactive protein concentrations, varies widely depending on factors such as parity, physiological status, management, and time after calving [15,16,17]. Review studies also point out that bioactive components such as lactoferrin may affect colostrum quality in ways that are not directly captured by standard indicators that mainly reflect immunoglobulin content [18,19]. Although % Brix and specific gravity are widely used, they were developed mainly as proxy measures for IgG concentration. Direct quantitative relationships between these on-farm indicators and other components of innate immunity, such as lactoferrin, remain limited [3,4,10,11,15,18]. In addition, most studies have used immunological assays, whereas LC–MS/MS methods allow precise and highly specific quantification of individual proteins in complex biological samples [20].

The aim of this pilot study was to track changes in lactoferrin concentration in bovine colostrum during the first 12 h after calving and to examine the relationship between lactoferrin concentration and commonly used on-farm colostrum quality indicators, including % Brix and specific gravity. We hypothesized that lactoferrin concentration decreases during the early postpartum period and that it may be associated with refractometric and density-based indicators of colostrum quality. The study also aimed to evaluate the rapid changes in colostrum quality during the first hours after calving.

## 2. Materials and Methods

### 2.1. Animals and Sampling

The study was conducted on a commercial dairy farm using six multiparous Holstein–Friesian cows, including second- (n = 2) and third-lactation (n = 4) animals that calved within the same period (December 2025). The cows were selected based on a comparable production level during the early postpartum period (approximately 20–22 L of milk within the first 12 h after calving) and similar health status during previous lactations. All cows were clinically healthy and had no history of clinical or subclinical mastitis during the previous lactation or at the time of sampling. All animals were maintained under identical housing and feeding conditions throughout the study period. Colostrum samples were collected at the first milking immediately after calving (0 h), and a second sampling was performed 12 h postpartum from the same animals, resulting in a total of 12 colostrum samples. The 12 h sampling interval corresponded to the standard farm practice for colostrum feeding of neonatal calves.

### 2.2. On-Farm Colostrum Analysis

All colostrum samples were analyzed on site at the farm to determine specific gravity using a hydrometer-type colostrometer (Colostrum Densimeter, KRUUSE A/S, Langeskov, Denmark). This device estimates colostrum quality based on its specific density and a color-calibrated scale for indirect assessment of immunoglobulin G (IgG) concentration.

Total soluble solids content was determined using a Brix refractometric method with a handheld colostrum refractometer (ColoQuick refractometer, Calvex A/S, Skive, Denmark), with a measurement range of 0–32% Brix and automatic temperature compensation (ATC).

### 2.3. Sample Storage and Laboratory Analysis

All colostrum samples (2 mL) were stored under controlled temperature conditions until laboratory analysis, at −80 °C. The determination of lactoferrin concentration was performed using liquid chromatography coupled with mass spectrometry (LC–MS/MS), according to the described analytical procedure. The HPLC-grade reagents used for lactoferrin analysis included ammonium bicarbonate (Carlo Erba, Val-de-Reuil, France), dithiothreitol (DTT; Sigma-Aldrich, St. Louis, MO, USA), and iodoacetamide (IAA; BLD Pharm, Reinbek, Germany). Bovine lactoferrin from colostrum (≥85% purity) was obtained from Sigma-Aldrich (St. Louis, MO, USA). Additional reagents included formic acid for LC–MS/MS analysis (≈98% purity; Honeywell, Seelze, Germany), acetonitrile (CHROMASOLV^®^, LC-MS grade, ≥99.9%; Honeywell, Seelze, Germany), and LC-MS-grade water (LiChrosolv^®^, Merck KGaA, Darmstadt, Germany). Donkey milk was used as a control matrix, obtained from a farm in the region of Sliven, Bulgaria. Trypsin (sequencing grade, modified, 4 × 25 μg) was purchased from SERVA (Heidelberg, Germany). The peptide internal standard ETTVFENLPEK (IS), containing a stable isotope-labeled amino acid ([^13^C_6_, ^15^N_2_]-lysine; purity > 98%), was obtained from Biomed Future Ltd. (Sofia, Bulgaria). The IS stock solution (2 mM) was stored at −80 °C and prepared in 20% acetonitrile in ultrapure water (Evoqua Water Technologies, Pittsburgh, PA, USA). All chemical reagents were dissolved in 100 mM ammonium bicarbonate.

### 2.4. LC–MS/MS Analysis of Lactoferrin

Lactoferrin analysis was performed according to a published LC–MS/MS method [20]. Donkey milk, without bovine lactoferrin, was used as a control (blank sample) and as a matrix for sample dilution. Standard solutions of bovine lactoferrin (1, 5, 10, 20, 50, and 100 nM) were prepared from a stock solution with a concentration of 1 mM in donkey milk as a matrix, immediately before use. Colostrum samples collected at 0 h and 12 h after calving were stored at −80 °C until analysis. The samples were diluted with ultrapure water to a final total protein concentration of approximately 10 mg mL^−1^. To aliquots of 200 μL, 20 μL of 1 μM internal standard were added, followed by the addition of 100 μL of 100 mM DTT. The samples were incubated at 60 °C for 1 h. Then, 200 μL of 100 mM IAA was added and the samples were again incubated at 30 °C for 30 min in the dark with constant shaking (1000 rpm; Heidolph Shakers & Mixers, Schwabach, Germany). Protein digestion was performed by adding 200 μL of trypsin solution (1 mg/mL in 25 mM ammonium bicarbonate) and incubating the samples overnight at 37 °C in the dark with constant shaking (1000 rpm). The reaction was stopped by adding 200 μL of 10% formic acid in water (*v*:*v*). The samples were centrifuged at 12,000× *g* for 10 min at 4 °C, and the supernatant was filtered through 0.22 μm filters (Agilent Captiva Econo Filter, PTFE membrane, Santa Clara, CA, USA) and transferred to LC–MS/MS vials.

The LC–MS/MS analysis was performed using an Agilent ZORBAX SB-C18 column (50 mm × 2.1 mm, particle size 1.8 μm), maintained at 40 °C, and an Agilent 6460 triple quadrupole mass spectrometer equipped with Jet Stream technology, a 1260 Infinity II quaternary pump, and a 1260 Infinity II vial sampler (Agilent Technologies, Santa Clara, CA, USA). The mobile phases included 0.1% formic acid in water (A) and 0.1% formic acid in acetonitrile (B), delivered at a flow rate of 0.4 mL/min with a gradient program. The injection volume was 5 μL. All other LC–MS/MS parameters were set according to Yuan et al. (2017) [20].

### 2.5. Statistical Analysis

All data were tested for normality using the Shapiro–Wilk test. Due to non-normal distribution and the paired nature of the samples, the non-parametric Wilcoxon signed-rank test was used to assess the effect of time on colostrum quality parameters. Statistical significance was accepted at *p* < 0.05. To evaluate the relationships between the studied parameters, linear regression analyses were performed for measurements obtained at 0 and 12 h postpartum. In the analysis, the dependent variable was lactoferrin concentration, and the independent variables were colostrum specific gravity and Brix. The results of the regression analysis are presented as coefficients (β), standard error (SE), t-value, and *p*-value, and graphs with a regression line and 95% confidence interval were prepared for visualization. GraphPad Prism (version 10.6.1) and JASP (Version 0.95.2, stats.org) were used for statistical analysis.

## 3. Results

### 3.1. Data Distribution (Shapiro–Wilk Test)

The normality of data distribution for each parameter and each time point was assessed using the Shapiro–Wilk test (Table 1). Lactoferrin concentration at 12 h after calving showed a statistically significant deviation from normal distribution (W = 0.5980; *p* < 0.001), whereas at 0 h no significant deviation from normality was detected (W = 0.8833; *p* = 0.285). Colostrum specific gravity, determined by a colostrometer, showed a statistically significant deviation from normal distribution at 0 h (W = 0.7744; *p* = 0.034), whereas at 12 h no significant deviation from normality was detected (W = 0.8744; *p* = 0.244). % Brix values did not show statistically significant deviation from normal distribution either at 0 h (W = 0.9287; *p* = 0.570) or at 12 h (W = 0.9048; *p* = 0.403).

### 3.2. Dynamics of Lactoferrin and Colostrum Quality Indicators Between 0 and 12 h After Calving

**Figure 1:**
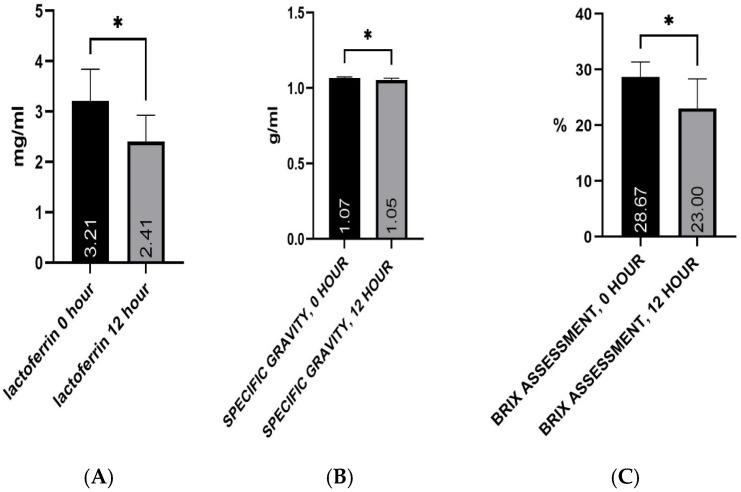
(**A**–**C**). Changes in colostrum quality parameters between 0 and 12 h postpartum. (**A**) Lactoferrin concentration. (**B**) Colostrum specific gravity. (**C**) Brix assessment. Data are presented as median values. * *p* < 0.05 (Wilcoxon signed-rank test).

**Figure 2:**
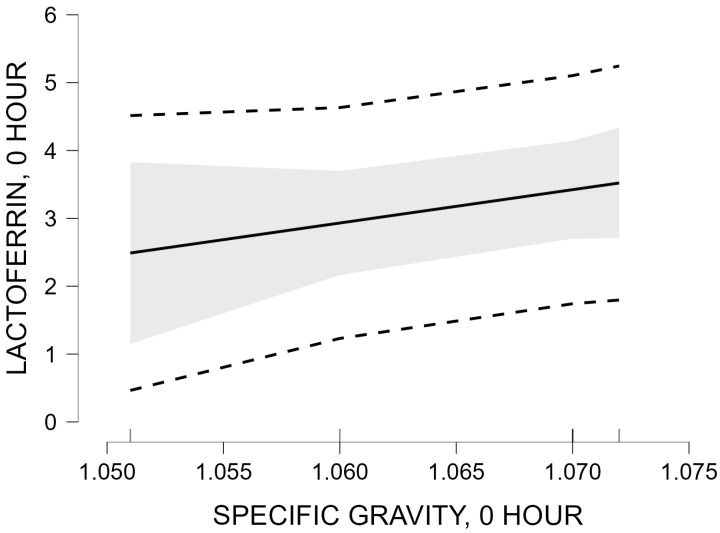
Marginal effect of colostrum specific gravity at 0 h on lactoferrin concentration at 0 h.

**Figure 3:**
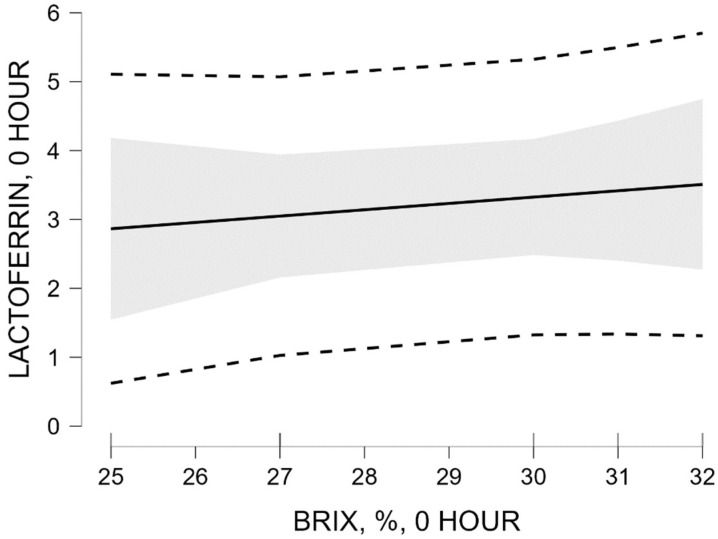
Marginal effect of Brix (%) at 0 h on lactoferrin concentration at 0 h.

Changes in lactoferrin concentration, colostrum specific gravity, and % Brix values between 0 and 12 h after calving were assessed using the non-parametric Wilcoxon signed-rank test (Figure 1, Figure 2 and Figure 3; Table 2).

Lactoferrin concentration decreased statistically significantly between 0 and 12 h after calving (Figure 1A; Wilcoxon signed-rank test, *p* = 0.031). The median lactoferrin concentration was 3.350 mg/mL at 0 h and 2.175 mg/mL at 12 h, with a median individual change between the two time points of −0.620 mg/mL. Colostrum specific gravity also showed a statistically significant decrease between 0 and 12 h after calving (Figure 1B; Wilcoxon signed-rank test, *p* = 0.031). The median specific gravity was 1.070 at 0 h and 1.056 at 12 h, with a median difference of −0.007. % Brix values decreased statistically significantly between 0 and 12 h after calving (Figure 1C; Wilcoxon signed-rank test, *p* = 0.031). The median % Brix values were 28.50% at 0 h and 23.00% at 12 h, with a median difference of −6.50 percentage points. Changes in lactoferrin concentration, colostrum specific gravity, and % Brix values between 0 and 12 h after calving are presented in Table 2.

For lactoferrin, the median decreased from 3.350 mg/mL at 0 h to 2.175 mg/mL at 12 h, with a median difference of −0.6200 mg/mL. For specific gravity, the median decreased from 1.070 at 0 h to 1.056 at 12 h, with a median difference of −0.0070. For % Brix, the median decreased from 28.50% at 0 h to 23.00% at 12 h, with a median difference of −6.500. For all parameters, the sum of positive ranks was 0 and the sum of negative ranks was −21, reflecting a consistent change toward lower values at 12 h compared with 0 h.

### 3.3. Linear Regression Between Lactoferrin Concentration and Colostrum Specific Gravity at 0 h

Linear regression analysis was used to assess the relationship between lactoferrin concentration (dependent variable) and colostrum specific gravity at 0 h (independent variable).

#### 3.3.1. Regression Coefficients

The linear regression coefficients, including unstandardized and standardized coefficients, standard errors, t-values, and *p*-values, are presented in Table 3.

The unstandardized regression coefficient for specific gravity was 49.127 (SE = 29.509), with a standardized coefficient β = 0.640. The slope test was not statistically significant (t = 1.665; *p* = 0.171).

#### 3.3.2. Model Fit and Explained Variance

Model fit indicators, including the coefficient of determination (R^2^), adjusted R^2^, and overall model significance, are presented in Table 4.

Regression model M_1_ explains 40.9% of the variation in lactoferrin concentration (R^2^ = 0.409; adjusted R^2^ = 0.262). The overall model significance was not statistically significant (*p* = 0.171).

#### 3.3.3. Marginal Effects Visualization

Visualization of the predicted relationship between colostrum specific gravity and lactoferrin concentration, as well as the 95% confidence interval, is presented in Figure 2.

The figure illustrates the positive slope of the regression line between colostrum specific gravity and the predicted lactoferrin concentration. The wide 95% confidence interval reflects substantial uncertainty in the effect estimate, consistent with the lack of statistical significance of the regression model. The results of the linear regression analysis at 0 h show a trend toward a positive relationship between colostrum specific gravity and lactoferrin concentration, which is not statistically significant (*p* = 0.171).

### 3.4. Linear Regression Between Lactoferrin Concentration and % Brix at 0 h

Linear regression analysis was used to assess the relationship between lactoferrin concentration (dependent variable) and % Brix values at 0 h (independent variable).

#### 3.4.1. Regression Coefficients

The linear regression coefficients, including unstandardized and standardized coefficients, standard errors, t-values, and *p*-values, are presented in Table 5.

The unstandardized regression coefficient for % Brix was 0.092 (SE = 0.107), with a standardized coefficient β = 0.395. The slope test was not statistically significant (t = 0.859; *p* = 0.439).

#### 3.4.2. Model Fit and Explained Variance

Model fit indicators for the regression model are presented in Table 6.

Regression model M_1_ explains 15.6% of the variation in lactoferrin concentration (R^2^ = 0.156; adjusted R^2^ = −0.055). The overall model significance was not statistically significant (*p* = 0.439).

#### 3.4.3. Marginal Effects Visualization

Visualization of the predicted relationship between % Brix values and lactoferrin concentration, as well as the 95% confidence interval, is presented in Figure 3.

The figure illustrates the positive slope of the regression line between % Brix values and the predicted lactoferrin concentration. The wide 95% confidence interval reflects substantial uncertainty in the effect estimate, consistent with the lack of statistical significance of the regression model. The results of the linear regression analysis at 0 h show a trend toward a positive relationship between % Brix values and lactoferrin concentration, which is not statistically significant (*p* = 0.439).

### 3.5. Linear Regression Between Lactoferrin Concentration and % Brix at 12 h

#### 3.5.1. Regression Coefficients

The linear regression coefficients, including unstandardized and standardized coefficients, standard errors, t-values, and *p*-values, are presented in Table 7.

The unstandardized regression coefficient for % Brix at 12 h was 0.062 (SE = 0.038), with a standardized coefficient β = 0.637. The slope test was not statistically significant (t = 1.651; *p* = 0.174).

#### 3.5.2. Model Fit and Explained Variance

Model fit indicators for the regression model are presented in Table 8.

Regression model M_1_ explains 40.5% of the variation in lactoferrin concentration (R^2^ = 0.405; adjusted R^2^ = 0.257). The overall model significance was not statistically significant (*p* = 0.174).

#### 3.5.3. Marginal Effects Visualization

**Figure 4:**
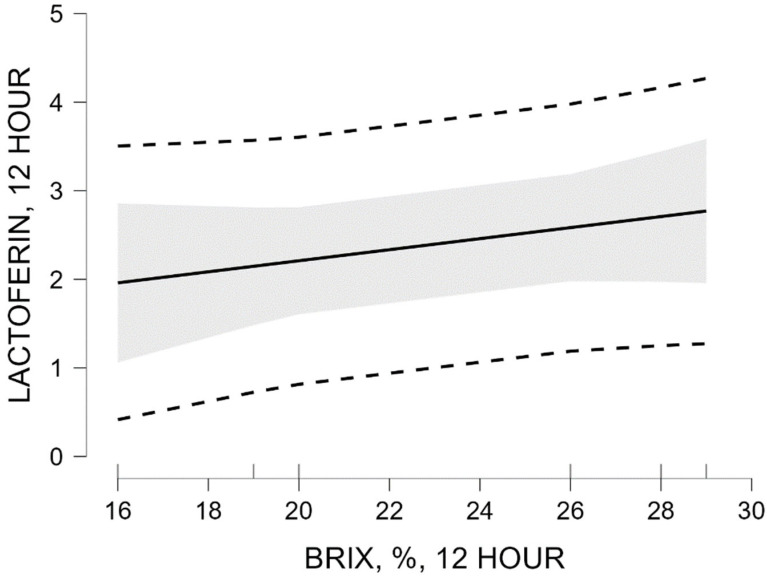
Marginal effect of Brix (%) at 12 h on lactoferrin concentration at 12 h.

Visualization of the predicted relationship between % Brix values and lactoferrin concentration, as well as the 95% confidence interval, is presented in Figure 4.

The figure illustrates the positive slope of the regression line between % Brix values and the predicted lactoferrin concentration. The wide 95% confidence interval reflects substantial uncertainty in the effect estimate, consistent with the lack of statistical significance of the regression model. The results of the linear regression analysis at 12 h show a trend toward a positive relationship between % Brix values and lactoferrin concentration, which is not statistically significant (*p* = 0.174).

### 3.6. Correlation Analysis (Spearman)

To assess the monotonic relationship between lactoferrin concentration and colostrum quality indicators, Spearman correlation analysis was performed at 0 and 12 h (Table 9).

Spearman correlation analysis did not identify statistically significant correlations between lactoferrin concentration and any of the studied colostrum quality indicators at 0 and 12 h (*p* > 0.05 for all comparisons). Nevertheless, all correlation coefficients were positive and of moderate magnitude (r = 0.55–0.70), reflecting the presence of trends toward positive association without reaching statistical significance.

Lactoferrin concentration in colostrum decreased statistically significantly between 0 and 12 h after calving, in parallel with a significant decrease in specific gravity and % Brix values. Linear regression analyses did not identify statistically significant relationships between lactoferrin concentration and any of the rapid colostrum quality indicators (specific gravity and % Brix) at either 0 or 12 h (*p* > 0.05 for all models), despite the presence of consistent positive slopes of the regression lines.

## 4. Discussion

The present study provides a quantitative assessment of the dynamics of lactoferrin concentration in colostrum during the first 12 h after calving and examines its relationship with widely used on-farm indicators of colostrum quality—specific gravity and % Brix. The results show a clear time dependence of all the studied parameters, as well as consistent but statistically non-significant positive associations between lactoferrin and indicators of total soluble solids content.

### 4.1. Dynamics of Lactoferrin and Colostrum Quality Indicators

The statistically significant decrease in lactoferrin concentration between 0 and 12 h after calving (median from 3.350 to 2.175 mg/mL; *p* = 0.031) is consistent with the rapid transition of colostrum composition toward transitional milk. The parallel decrease in specific gravity (from 1.070 to 1.056) and % Brix values (from 28.50% to 23.00%) is in agreement with the decline in protein concentration and other soluble solids during the early postpartum period, as reported in previous studies [1,2]. These findings indicate that colostrum undergoes rapid quantitative changes within the first hours after calving. While most previous studies have focused primarily on immunoglobulin G as the main component of colostral immunity, the present data suggest that lactoferrin also follows a temporal pattern during the early postpartum period that coincides with the overall decline in colostral proteins. Similar patterns in the concentrations and yields of lactoferrin and other bioactive components of milk have been reported in recent studies examining colostrum and early lactation milk in dairy cattle [14].

### 4.2. Relationship Between Lactoferrin and Specific Gravity at 0 h After Calving

Linear regression analysis between lactoferrin concentration and specific gravity at 0 h showed a moderate coefficient of determination (R^2^ = 0.409; adjusted R^2^ = 0.262) and a positive standardized coefficient (β = 0.640), although the relationship did not reach statistical significance (*p* = 0.171). Approximately 41% of the variation in lactoferrin concentration was explained by variation in specific gravity within the model; however, this association was not statistically significant at the present sample size. This observation is consistent with the concept that specific gravity and refractometric indicators primarily reflect total soluble solids content, which is largely influenced by immunoglobulins [3,4,5]. Lactoferrin represents a smaller fraction of the total protein pool, and its contribution to overall specific gravity may therefore be limited. The moderate R^2^ value and positive β coefficient may reflect a potential relationship that could require confirmation in studies including larger numbers of animals.

### 4.3. Relationship Between Lactoferrin and % Brix at 0 and 12 h After Calving

At 0 h, the relationship between % Brix and lactoferrin concentration showed lower explained variance (R^2^ = 0.156) and a moderate standardized coefficient (β = 0.395), without statistical significance (*p* = 0.439). These results suggest that % Brix, although widely validated as an indirect indicator of IgG, may not reflect variation in lactoferrin concentration with high sensitivity. This observation is consistent with previous studies indicating that refractometry is primarily optimized for the assessment of immunoglobulin content rather than individual proteins. At 12 h after calving, the relationship between % Brix and lactoferrin showed higher explained variance (R^2^ = 0.405; adjusted R^2^ = 0.257) and a higher standardized coefficient (β = 0.637), again without statistical significance (*p* = 0.174).

Variability in colostrum composition and relationships among milk components have been described in recent studies of dairy cattle [21]. Recent work has also examined concentrations of lactoferrin and other bioactive components in colostrum and early lactation milk [14]. The higher R^2^ value at 12 h compared with 0 h may indicate that the relative contribution of lactoferrin to total soluble solids content becomes more apparent as colostrum composition changes during the early postpartum period. This observation may reflect differences in the temporal dynamics of lactoferrin and immunoglobulins during the transition from colostrum to transitional milk, as reported in studies examining protein profile changes during early lactation [15]. These findings suggest that % Brix should be interpreted primarily as an indicator of immunoglobulin-related soluble solids rather than as a universal indicator of all immunologically active proteins in colostrum.

### 4.4. Correlation Analysis and Biological Interpretation

Spearman correlation analysis did not identify statistically significant associations between lactoferrin and any of the studied indicators; however, all correlation coefficients were positive and of moderate magnitude (r = 0.55–0.70). This includes the relationships between lactoferrin and specific gravity at 0 h (r = 0.6983) and between lactoferrin and % Brix at 12 h (r = 0.6367). The consistency of positive coefficients across the analyses indicates a similar direction of association between lactoferrin and the studied indicators, although none of the correlations reached statistical significance. The combination of moderate R^2^ values, positive β coefficients, and moderate correlation coefficients may indicate a potential relationship between lactoferrin and indicators of colostrum quality that requires confirmation in studies including larger numbers of animals. Such patterns are commonly observed in pilot studies, where limited sample size restricts the statistical power to detect moderate associations.

### 4.5. Scientific and Practical Contribution

The results are consistent with the concept that refractometric and density-based indicators primarily reflect adaptive immune components dominated by immunoglobulins, whereas lactoferrin represents an additional component of innate immunity that may not be fully reflected by these rapid on-farm methods [12,13]. From a practical perspective, high % Brix values should therefore not be automatically interpreted as indicators of high lactoferrin concentrations. The use of LC–MS/MS for the quantitative determination of lactoferrin provides high analytical specificity and enables more detailed characterization of the bioactive components of colostrum [20]. The combination of LC–MS/MS analysis with on-farm colostrum quality indicators and evaluation of temporal changes during the early postpartum period represents an approach that may provide additional information on variation in the bioactive components of colostrum. The observed decrease in lactoferrin concentration during the first 12 h after calving and the positive but statistically non-significant relationships with specific gravity and % Brix provide additional information on the dynamics of bioactive proteins in early colostrum. These results suggest that standard on-farm methods used to assess colostrum quality may not fully reflect components of innate immunity and that lactoferrin may represent an additional indicator of colostrum composition. Previous studies have reported substantial variability in colostrum composition among dairy cows, indicating potential biological variation in colostral components beyond immunoglobulins [22]. Earlier work has also described changes in lactoferrin and related iron-binding proteins during lactation, which is consistent with the temporal patterns observed in the present study [23].

## 5. Limitations

The present study included a limited number of animals (*n* = 6), which limits the statistical power of the analysis and the generalization of the results. The study was designed as a preliminary investigation aimed at examining early postpartum changes in lactoferrin concentration in bovine colostrum. The sampling period was limited to the first 12 h after calving. This time frame was selected in order to investigate changes in colostrum composition during the early postpartum period, when colostrum secretion is most pronounced and rapid changes in its components occur. An additional limitation is the absence of direct measurements of immunoglobulin G and somatic cell counts, which does not allow direct comparison between components of innate and adaptive immunity in colostrum.

The study was conducted on animals from a single farm, which may limit the generalization of the results to other herds and management systems.

Future studies including a larger number of animals, extended sampling periods, and additional measurements of immunoglobulins in calf serum and other blood parameters of neonatal calves will allow a more comprehensive evaluation of the relationship between lactoferrin concentration and colostrum quality.

## 6. Conclusions

The present study examined the early dynamics of lactoferrin in bovine colostrum and its relationship with widely used on-farm quality indicators. A significant decrease in lactoferrin concentration was observed during the first 12 h after calving, reflecting rapid changes in colostrum composition during the early postpartum period. The lack of statistically significant relationships between lactoferrin and specific gravity or % Brix indicates that standard rapid on-farm methods may not directly reflect lactoferrin concentration. These findings suggest that colostrum quality assessment based solely on refractometric or density-based indicators may not fully capture variation in lactoferrin concentration. Quantitative determination of lactoferrin using LC–MS/MS in combination with parallel on-farm assessment and evaluation of temporal changes during the early postpartum period offers additional information on the relationship between lactoferrin concentration and commonly used colostrum quality indicators.

Further studies including larger numbers of animals and additional immunological indicators are required to better characterize the relationship between lactoferrin concentration and colostrum quality.

## Figures and Tables

**Table 1:** Shapiro–Wilk test for normality of data distribution.

Indicator	Period	W Value	*p* Value	Normality
Lactoferrin	0 h	0.8833	0.285	Yes
Lactoferrin	12 h	0.5980	<0.001 ***	No
Colostrometer	0 h	0.7744	0.034 *	No
Colostrometer	12 h	0.8744	0.244	Yes
Refractometer (Brix)	0 h	0.9287	0.570	Yes
Refractometer (Brix)	12 h	0.9048	0.403	Yes

Significance: * *p* < 0.05; *** *p* < 0.001.

**Table 2:** Changes in lactoferrin concentration, colostrum specific gravity and Brix assessment between 0 and 12 h postpartum and results of the Wilcoxon signed-rank test.

Indicator	n	Median 0 h	Median 12 h	Median of Difference	Sum of Positive Ranks	Sum of Negative Ranks	W (Signed Ranks)	*p* Value	Exact/Approximate	One-/Two-Tailed	Significance (*p* < 0.05)	Spearman rs (Pairing)
Lactoferrin (mg/mL)	6	3.350	2.175	−0.6200	0.000	−21.00	−21.00	0.031 *	Exact	Two-tailed	Yes	0.3714 (ns, *p* = 0.249)
Colostrometer (Specific gravity)	6	1.070	1.056	−0.0070	0.000	−21.00	−21.00	0.031 *	Exact	Two-tailed	Yes	0.0308 (ns, *p* = 0.492)
Refractometer (Brix)	6	28.50	23.00	−6.500	0.000	−21.00	−21.00	0.031 *	Exact	Two-tailed	Yes	0.7537 (ns, *p* = 0.056)

* *p* < 0.05 was considered statistically significant.

**Table 3:** Regression coefficients for the relationship between colostrum specific gravity at 0 h and lactoferrin concentration.

Model	R^2^	Unstandardized	Standard Error	Standardized (β)	t	*p*
M_0_ (Intercept)	0.000	3.202	0.260	—	12.322	<0.001
M_1_ (Intercept)	0.409	−49.143	31.443	—	−1.563	0.193
M_1_ SPECIFIC GRAVITY, 0 HOUR		49.127	29.509	0.640	1.665	0.171

**Table 4:** Model summary for the linear regression between colostrum specific gravity at 0 h and lactoferrin concentration.

Model	R	R^2^	Adjusted R^2^	RMSE	R^2^ Change	df1	df2	*p*
M_0_	0.000	0.000	0.000	0.636	0.000	0	5	
M_1_	0.640	0.409	0.262	0.547	0.409	1	4	0.171

Note: M_1_ includes SPECIFIC GRAVITY, 0 h.

**Table 5:** Regression coefficients for the relationship between Brix (%) at 0 h and lactoferrin concentration.

Model	R^2^	Coefficient	Standard Error	Standardized (β)	t	*p*
M_0_ Intercept	0.000	3.202	0.260	—	12.322	<0.001
M_1_ Intercept	0.156	0.565	3.079	—	0.184	0.863
M_1_ BRIX, %, 0 HOUR		0.092	0.107	0.395	0.859	0.439

**Table 6:** Model summary for the linear regression between Brix (%) at 0 h and lactoferrin concentration.

Model	R	R^2^	Adjusted R^2^	RMSE	R^2^ Change	df1	df2	*p*
M_0_	0.000	0.000	0.000	0.636	0.000	0	5	
M_1_	0.395	0.156	−0.055	0.654	0.156	1	4	0.439

**Note:** M_1_ includes BRIX, %, 0 HOUR.

**Table 7:** Regression coefficients for the relationship between Brix (%) at 12 h and lactoferrin concentration.

Model	R^2^	Coefficient	Standard Error	Standardized (β)	t	*p*
M_0_ Intercept	0.000	2.397	0.215	—	11.169	<0.001
M_1_ Intercept	0.405	0.962	0.888	—	1.084	0.339
M_1_ BRIX, %, 12 HOUR		0.062	0.038	0.637	1.651	0.174

**Table 8:** Model summary for the linear regression between Brix (%) at 12 h and lactoferrin concentration.

Model	R	R^2^	Adjusted R^2^	RMSE	R^2^ Change	df1	df2	*p*
M_0_	0.000	0.000	0.000	0.526	0.000	0	5	
M_1_	0.637	0.405	0.257	0.453	0.405	1	4	0.174

Note: M_1_ includes BRIX, %, 12 HOUR.

**Table 9:** Spearman correlation coefficients between lactoferrin and milk quality parameters at 0 and 12 h (*n* = 6).

Indicator Comparison	Test	r	*p* Value	*n*	Significance
Lactoferrin, 0 h vs. Specific gravity, 0 h	Spearman	0.6983	0.1500	6	ns
Lactoferrin, 0 h vs. Brix, 0 h	Spearman	0.5508	0.2722	6	ns
Lactoferrin, 12 h vs. Specific gravity, 12 h	Spearman	0.6377	0.2000	6	ns
Lactoferrin, 12 h vs. Brix, 12 h	Spearman	0.6367	0.1740	6	ns

Note: ns = not significant (*p* > 0.05).

## Data Availability

The original contributions presented in this study are included in the article. Further inquiries can be directed to the corresponding author.
